# Cetuximab PET delineated changes in cellular distribution of EGFR upon dasatinib treatment in triple negative breast cancer

**DOI:** 10.1186/s13058-020-01270-1

**Published:** 2020-04-15

**Authors:** Brooke N. McKnight, Seongho Kim, Julie L. Boerner, Nerissa T. Viola

**Affiliations:** 1 Department of Oncology, Karmanos Cancer Institute Wayne State University, 4100 John R Street, Detroit, MI 48201 USA; 2 Department of Oncology, Biostatistics Core, Karmanos Cancer Institute Wayne State University, Detroit, MI 48201 USA

**Keywords:** Nuclear EGFR translocation, EGFR PET imaging, Triple negative breast cancer, Src, Dasatinib

## Abstract

**Background:**

At least 50% of triple negative breast cancer (TNBC) overexpress the epidermal growth factor receptor, EGFR, which paved the way for clinical trials investigating its blockade. Outcomes remained dismal stemming from mechanisms of resistance particularly the nuclear cycling of EGFR, which is enhanced by Src activation. Attenuation of Src reversed nuclear translocation, restoring EGFR to the cell surface. Herein, we hypothesize that changes in cellular distribution of EGFR upon Src inhibition with dasatinib can be annotated through the EGFR immunopositron emission tomography (immunoPET) radiotracer, [^89^Zr]Zr-cetuximab.

**Methods:**

Nuclear and non-nuclear EGFR levels of dasatinib-treated vs. untreated MDA-MB-231 and MDA-MB-468 cells were analyzed via immunoblots. Both treated and untreated cells were exposed to [^89^Zr]Zr-cetuximab to assess binding at 4 °C and 37 °C. EGFR-positive MDA-MB-231, MDA-MB-468, and a patient-derived xenograft were treated with dasatinib or vehicle followed by cetuximab PET imaging to compare EGFR levels. After imaging, the treated mice were separated into two groups: one cohort continued with dasatinib with the addition of cetuximab while the other cohort received dasatinib alone. Correlations between the radiotracer uptake vs. changes in tumor growth and EGFR expression from immunoblots were analyzed.

**Results:**

Treated cells displayed higher binding of [^89^Zr]Zr-cetuximab to the cell membrane at 4 °C and with greater internalized activity at 37 °C vs. untreated cells. In all tumor models, higher accumulation of the radiotracer in dasatinib-treated groups was observed compared to untreated tumors. Treated tumors displayed significantly decreased pSrc (Y416) with retained total Src levels compared to control. In MDA-MB-468 and PDX tumors, the analysis of cetuximab PET vs. changes in tumor volume showed an inverse relationship where high tracer uptake in the tumor demonstrated minimal tumor volume progression. Furthermore, combined cetuximab and dasatinib treatment showed better tumor regression compared to control and dasatinib-only-treated groups. No benefit was achieved in MDA-MB-231 xenografts with the addition of cetuximab, likely due to its KRAS-mutated status.

**Conclusions:**

Cetuximab PET can monitor effects of dasatinib on EGFR cellular distribution and potentially inform treatment response in wild-type KRAS TNBC.

## Background

Triple negative breast cancer (TNBC) accounts for 20% of all diagnosed breast cancers. Its lack of biomarkers (ER/PR/HER2) makes it more difficult to treat, with chemotherapies such as taxane or anthracycline as the mainstay standard of care [1]. While many patients initially respond to chemotherapy, the high rate of recurrence makes it a far more aggressive disease with worse prognosis compared to other subtypes. This created a strong impetus to find other biomarkers for targeting TNBC. Gene expression profiling studies identified the epidermal growth factor receptor (EGFR) as a potential biomarker in at least 50% of patients with TNBC [2], paving the way for clinical trials investigating EGFR-targeted therapies, including monoclonal antibodies (panitumumab and cetuximab) and small molecule inhibitors (gefitinib, erlotinib, and afatinib) within this patient population [3]. Unfortunately, early clinical data reported dismal response rates [4, 5]. One rationale likely stems from the loss of EGFR from the cell surface as a result of its translocation to the nucleus; thus, potentially diminishing drug target and accessibility.

Studies have demonstrated that nuclear EGFR (nEGFR) acts as a transcription factor regulator involved in tumorigenesis [6, 7], is associated with poorer outcomes in many cancers [8–10], and is implicated in resistance to radiation and anti-EGFR therapies, including cetuximab [11, 12]. Transport of EGFR to the nuclear regions was reported to be mediated through Src hyperactivation [13–15]. Previous studies have shown that blockade of Src kinase activity halts nEGFR translocation and increases its accumulation in the plasma membrane, which enhanced cetuximab sensitivity in non-small cell lung cancer (NSCLC) and TNBC [11, 12, 16].

With this in mind, we hypothesize that monitoring changes in EGFR distribution within the cellular milieu post-Src targeted inhibition with dasatinib, an FDA-approved Src/Bcr/ABL inhibitor, can be monitored by EGFR immunopositron emission tomography (immunoPET). In this study, we utilized zirconium-89 (*t*_1/2_ ~ 3.27 days)-labeled cetuximab (Erbitux®) as our EGFR-specific immunoPET tracer. To date, [^89^Zr]Zr-cetuximab has shown promise in visualizing tumors expressing EGFR and could be used to monitor EGFR expression and steer individualized treatments in the clinic [17]. We first tested its specificity in established TNBC cell lines with EGFR overexpression, namely MDA-MB-231 (KRAS mutant), MDA-MB-468 (KRAS wild type (wt)), and the low EGFR-expressing MDA-MB-453 (KRAS wild type). We assessed the binding and uptake of the tracer through in vitro assays using MDA-MB-231 and MDA-MB-468 that were treated with the derived half maximal inhibitory concentration (IC_50_) of dasatinib and compared it against EGFR immunoblots for nuclear and non-nuclear (cytosolic plus membranous) EGFR. We next examined our hypothesis in a longitudinal study using established MDA-MB-231, MDA-MB-468 and TNBC patient-derived xenografts (PDX). We compared the uptake of [^89^Zr]Zr-cetuximab in dasatinib-treated vs. control untreated tumor-bearing mice and monitored tumor regression. After imaging, the treatment arms were further separated into dasatinib only and dasatinib plus cetuximab treatment groups. Correlations to tumor regression and protein expression were analyzed. Ex vivo western blots were analyzed to confirm the measured uptake of [^89^Zr]Zr-cetuximab across all tumor models.

## Materials and methods

### Cell lines and reagents 

MDA-MB-231 cells were a generous gift from Prof. Stephan Patrick at Wayne State University (WSU). All cell lines (ATCC) were grown in 5% CO_2_ using DMEM supplemented with 1% penicillin-streptomycin and 5% fetal bovine serum (Sigma) as media at 37 °C. All cells were manipulated in a sterile environment and routinely tested for mycoplasma with MycoAlert Mycoplasma Detection Kit (Lonza) and certified by the Biobanking and Correlative Services Core at WSU. Dasatinib (Selleckchem) was prepared as a 30 mM stock in dimethyl sulfoxide (DMSO). Cetuximab is commercially available and was obtained from the Karmanos Cancer Center pharmacy. All other materials and reagents used are listed in the supplemental information provided.

### In vivo tumor models

All animal handling and manipulations were conducted in accordance with the guidelines set by WSU Institutional Animal Use and Care Committee. Mice were given food and water *ad libitum*. For imaging experiments, female athymic nu/nu mice (6–8 weeks old, Envigo) were subcutaneously (s.c.) injected with either 2 × 10^6^ MDA-MB-468 (KRAS wt), 5 × 10^6^ MDA-MB-231 (KRAS mutant), or 5 × 10^6^ MDA-MB-453 (KRAS wt) BC cells. Cells were injected as a suspension in 150 μL 1:1 media: Matrigel (BD Biosciences, Bedford, MA) on the right shoulder. For the PDX model (KRAS wt), female SCID mice (6–8 weeks old, Envigo) were implanted with freshly sliced tumor pieces (Jackson Labs, Model ID no. TM00089 Primary Invasive Ductal Carcinoma) washed in PBS and then dipped in Matrigel before implantation with a trocar. Tumor growth was monitored weekly with calipers. The tumor volume was calculated using the formula: length × width × height × *π*/6. Mice with tumor volumes ranging from 150 to 250 mm^3^ were utilized for xenograft studies, and 50–100 mm^3^ were utilized for PDX studies.

### Radiosynthesis of [^89^Zr]Zr-cetuximab

p-Benzyl-isothiocyanate-desferrioxamine (DFO, Macrocylics, Inc.) was conjugated to cetuximab according to published protocols [18]. The synthesis was performed using 5:1 mole equivalence of DFO to cetuximab in 0.9% saline, pH ~ 9 at 37 °C for 1 h. The pure, monoclonal antibody (mAb) DFO-conjugate was obtained by passing through a spin column filter with a molecular weight cut-off of 30 kDa (GE Vivaspin 500) using sterile saline as eluting buffer.

Approximately 1 mCi (37 MBq) of [^89^Zr]Zr-oxalate (3D Imaging, LLC) was neutralized to pH 7.0–7.2 using 1 M NaOH. Cetuximab-DFO (200 μg) was added to the ^89^Zr solution. The reaction was quenched after 1–1.5-h incubation at room temperature upon addition of 5 μL of 50 mM EDTA (pH ~ 7) to eliminate any non-specifically bound ^89^Zr. A radiolabeling efficiency of > 95% was determined via radio-instant thin layer chromatography (radio-iTLC) using a silica gel-impregnated strip (Agilent Technologies, Santa Clara, CA) and 50 mM EDTA as the solid and mobile phase, respectively. Pure [^89^Zr]Zr-cetuximab was obtained through spin column centrifugation (GE Vivaspin 500, MWCO: 30 kDa) with sterile saline used for eluting unbound radiometal. A radiochemical purity of > 95% was obtained based on radio-iTLC analysis. [^89^Zr]Zr-cetuximab was assessed for immunoreactivity as previously described [19].

### IC_50_ calculations

IC_50_ values were obtained for MDA-MB-468 and MDA-MB-231 breast cancer cell lines. Wells were seeded with ~ 1 × 10^4^ cells and incubated overnight at 37 °C in 5% CO_2_. Cells were treated with increasing concentrations of dasatinib (1 nM to 1 mM) and incubated for 72 h then analyzed for viability using Alamar blue assay (Life Technologies). After 4-h incubation with Alamar blue, absorbance was read at 570 nm on an Infinite M200 plate reader (Tecan). The control well was subtracted from each treatment well, and the IC_50_ was calculated as log [dasatinib] vs. normalized response.

### In vitro binding assay

Internalization of [^89^Zr]Zr-cetuximab was evaluated on MDA-MB-468, MDA-MB-231, and MDA-MB-453 TNBC cell lines. Wells were seeded with ~ 5 × 10^4^ cells and incubated overnight. Cells were treated with the established IC_50_ for dasatinib in complete media for 48 h. After incubation, [^89^Zr]Zr-cetuximab (150 ng, 0.75 μCi, 27.75 kBq) in 1 mL of media was added to each well. The plates were incubated at either 4 °C or 37 °C for 2 h. Following the incubation period, the media was collected, and the cells were rinsed with 1× phosphate-buffered saline (PBS) twice. Surface-bound activity was removed by washing the cells in 100 mM acetic acid + 100 mM glycine in PBS (1:1, pH 3.5) at 4 °C. The cells were then lysed with 1 M NaOH. All washes (media plus PBS, acid and alkaline) were collected in separate tubes and measured for bound activity using a gamma counter (Perkin Elmer). The percentage of internalized activity was calculated as the ratio of the activity of the lysate and the total activity collected from the media, PBS, acid and base washes, normalized to 50,000 cells counted using a Countess II Automated Cell Counter (Thermo Fisher).

### Western blotting

Lysates were prepared in NuPAGE LDS sample buffer (Life Technologies) supplemented with 2-mercaptoethanol (Sigma-Aldrich), and boiled at 95 °C for 5 min. Proteins and ladder were separated on a 4–12% bis-tris gel before transfer to Immobilon-P PVDF membrane (Millapore Sigma). Membranes were blocked in 5% non-fat dry milk in TBST for 1 h at room temperature. Primary antibodies (Supplemental Information) were diluted 1:1000 in TBST with 0.02% sodium azide and incubated at 4 °C overnight with gentle rocking. Secondary antibodies (1:1000) were incubated at room temperature for 2 h in 5% milk-TBST. Proteins were visualized using Amersham ECL (GE), and images were collected using a ChemiDoc (BioRad) system. Images were analyzed and quantified using Image Lab (BioRad) software. The protein to GAPDH and protein to H3 ratios were obtained via densitometric analysis using ImageJ. Additional detailed methods can be found in the supplementary information.

### Mouse treatment studies

Dasatinib (50 mg/kg dose dissolved in 150 μL 1:1 sterile water:propylene glycol) was administered to MDA-MB-231 (*n* = 10) and MDA-MB-468 (*n* = 10) tumor-bearing mice via oral gavage for 5 days prior to PET imaging. Untreated control mice (*n* = 5) were given the vehicle. After PET imaging, dasatinib-treated mice were separated into two groups. One cohort (*n* = 5) received additional cetuximab intraperitoneally (i.p.) (0.3 mg 2×/week for 3 weeks) in combination with dasatinib (50 mg/kg). The second group was continued on dasatinib treatment alone (*n* = 5). The control group remained untreated. PDX tumors were given dasatinib followed by the combination treatment after PET imaging. Tumor volumes were recorded once per week. The percentage of change in tumor volume was obtained using volumes measured at the start and on the final day of treatment before mice were euthanized.

### PET imaging

Tumor-bearing mice were injected with [^89^Zr]Zr-cetuximab [180–200 μCi, 6.6–7.4 MBq, 36–40 μg, 2.4–2.7 nmol] in sterile saline on the lateral tail vein. Small-animal PET scans were acquired from 24 to 96 h p.i. using a Focus 220 scanner (Concorde Microsystems), establishing 48 h p.i. as the optimal time point. The mice were fully anesthetized with 1–2% isoflurane (Baxter, Deerfield, IL) during each scan. Images were reconstructed via filter back projection and decay-corrected to the time of injection prior to analysis. Volumes of interest (VOI) expressed as the mean percentage of injected dose per gram of tissue (%ID/g) were obtained on various planar sections by manually drawing on the tumor and on select organs using ASIPro VM™ v.6.3.3.0 software (Concorde Microsystems).

### Ex vivo validation

Tumors were excised on the last day of imaging for ex vivo validation through autoradiography and western blot. Autoradiography was performed following previously reported protocols [20]. Briefly, tumors excised post-imaging were snap frozen in liquid nitrogen before being embedded in OCT medium (Fisher) and then frozen in dry ice. After blocks were completely frozen, tumors were cut into 5-μm sections (Leica CM 1850) and mounted on positively charged slides (Fisher). Digital autoradiography was performed by placing slides in a film cassette against a phosphor imaging plate (Fujifilm BAS-MS2325, Fuji Photo Film) at − 20 °C overnight. Phosphor imaging plates were read at a pixel resolution of 25 μm with a Typhoon 7000 IP plate reader (GE Healthcare).

For western blot, tumors were decayed to background for at least 10 half-lives. Western blots were conducted on tumor lysates according to the protocol stated above.

### Statistical analysis

Data were analyzed using Graphpad Prism v. 7.02 unless otherwise stated. Statistical analysis was performed using one-way ANOVA test in in vitro uptake assays. Tumor uptake and western blot comparisons were performed using unpaired *t* test. Non-parametric, two-tailed Spearman correlation was used to assess the relationship between tumor uptake in VOI vs. (i) total EGFR/GAPDH and (ii) volume change. The tumor growth rates over time were compared using linear mixed-effects models on log-transformed data, and the *p* values were obtained using Wald tests. The overall tumor volume over time was computed based on the area under the tumor growth curve (AUC) that was normalized by day. The overall tumor volumes were compared using unpaired *t* tests on log-transformed normalized AUC. The *p* values were adjusted for multiplicity by the Holm’s procedure. A value of *p* < 0.05 was considered statistically significant. Data were expressed as the mean ± S.D.

## Results

### Radiolabeling and characterization of [^89^Zr]Zr-cetuximab

Cetuximab radiolabeling yields of > 90% were obtained with > 95% purity after purification via spin column. A specific activity of 4.7 ± 0.3 mCi/mg was achieved. The labeled antibody retained immunoreactivity towards EGFR with 74.8 ± 3.4% (Fig. S1A, *n* = 3).

### Effects of dasatinib treatment in vitro

**Fig. 1:**
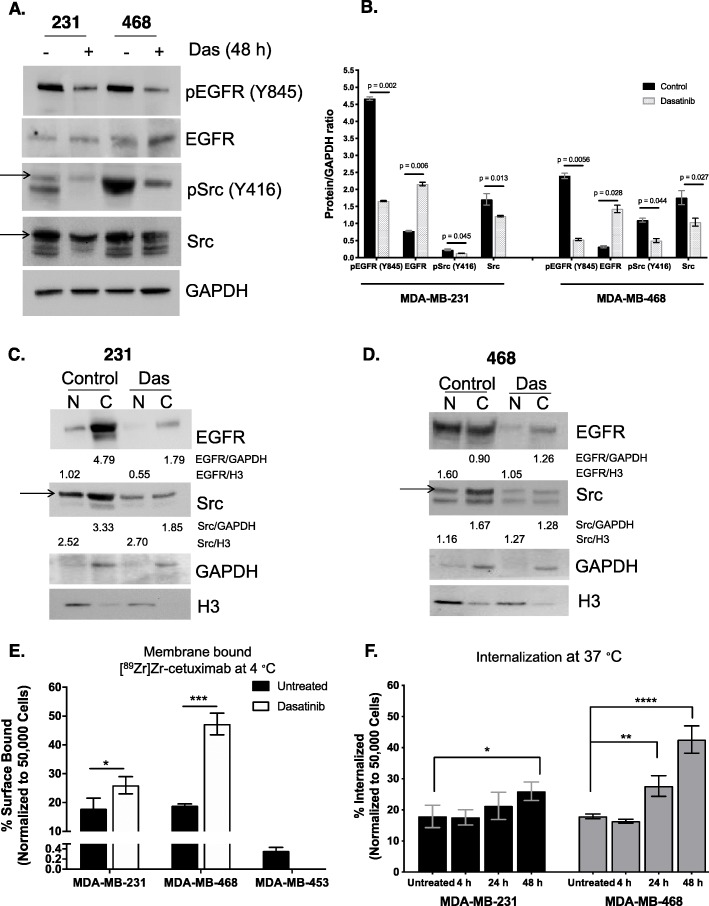
In vitro dasatinib (Das) treatment alters EGFR compartmentalization. MDA-MB-231 (left) and MDA-MB-468 (right) cells were treated with dasatinib IC_50_ values for 48 h (+) or left untreated (−). A representative western blot shows expression of pEGFR (Tyr845), EGFR, pSrc (Tyr416), and Src (band identified by an arrow) (**a**). A plot of densitometry values of untreated and treated cell lysates (**b**). Densitometry values were obtained as the ratio of protein to GAPDH. Nuclear (N) and membrane plus cytoplasmic (C) extracts were collected from MDA-MB-231 (**c**) and MDA-MB-468 (**d**) cells after 48 h with and without treatment and evaluated for EGFR and Src expression and activity. Protein derived from nuclear compartment was normalized against H3 loading control while protein from the cytoplasm was normalized against GAPDH. High membrane-bound [^89^Zr]Zr-cetuximab was displayed in treated versus untreated cells incubated at 4 °C to stop temperature-dependent internalization (**e**). Higher internalized [^89^Zr]Zr-cetuximab is displayed at 37 °C in the treated groups (**f**)

We first sought to evaluate cellular response to dasatinib in vitro by observing protein expression changes in EGFR and Src after treatment. After exposing cells to dasatinib for 48 h, IC_50_ values of 0.88 ± 0.10 μM (Fig. S1B) and 19.30 ± 0.06 μM (Fig. S1C) were determined for MDA-MB-231 and MDA-MB-468 cells, respectively, and further used for all in vitro assays. In treated MDA-MB-231 cell lysates (Fig. 1a, Table S1) incubated with dasatinib for 48 h, a decrease in pEGFR (Y845) from 4.67 ± 0.05 to 1.66 ± 0.02 (*p* = 0.0025) and pSrc (Y416) levels from 0.23 ± 0.02 to 0.13 ± 0.003 (*p* = 0.045) as measured by densitometry was observed (Fig. 1b). Total EGFR levels increased while Src expression decreased after treatment. In MDA-MB-468 cells (Fig. 1a, Table S1), a significant decrease was similarly displayed in both pEGFR (Y845) (2.40 ± 0.08 to 0.53 ± 0.03, *p* = 0.006) and pSrc (Y416) (1.10 ± 0.06 to 0.50 ± 0.06, *p* = 0.044) after treatment. Similar to MDA-MB-231, total EGFR levels significantly increased while Src expression was lower in the dasatinib-treated cells.

We next sought to determine the nuclear (N) and non-nuclear (membranous plus cytoplasmic, C) localization of EGFR after dasatinib treatment similar to the study by Brand et al. showing nuclear versus non-nuclear EGFR [12]. nEGFR decreased for both MDA-MB-231 (Fig. 1c) and MDA-MB-468 (Fig. 1d) lysates. EGFR protein in the cytosolic and membranous regions increased for both cell lines. Collectively, our in vitro data demonstrate concordance with previous reports, wherein mitigated Src activity (Fig. 1a, b) concomitantly decreased levels of nEGFR.

### In vitro binding assay with [^89^Zr]Zr-cetuximab

The internalization of EGFR upon dimerization [21, 22] or binding to antibodies [23, 24] was previously reported to be dependent on temperature, among other mechanisms. Since the antibody only binds to membrane-localized receptor, we investigated the potential of [^89^Zr]Zr-cetuximab to measure changes in surface levels of EGFR at 4 °C to halt internalization. Cells were exposed to dasatinib over 48 h at 37 °C followed by incubation with [^89^Zr]Zr-cetuximab at 4 °C. Increased membranous EGFR was detected by the radiotracer for both treated MDA-MB-231 (17.9 ± 3.6% vs. 26.0 ± 3.0%, *p* = 0.042) and MDA-MB-468 (18.9 ± 0.6% vs. 47.3 ± 3.8%, *p* < 0.001) cells compared to untreated groups (Fig. 1e). Cell-surface-bound fractions of the tracer in low EGFR-expressing MDA-MB-453 (untreated) was minimal.

We next examined differences in internalized [^89^Zr]Zr-cetuximab between control and treated groups at 37 °C and at different exposure times to prove that the increased membranous EGFR directly correlates to higher internalization (Fig. 1f). While no difference in membrane-associated radioactivity was exhibited (Fig. S1D) for both cell lines, an increase in internalization of the tracer after 48 h of drug treatment compared to control was observed in MDA-MB-231 cells (16.6 ± 3.3 vs. 26.2 ± 3.7, *p* < 0.001). Similar but more pronounced effects were observed with the treated MDA-MB-468 cells, wherein internalized fractions were higher by 1.5-fold (27.7 ± 3.33%, *p* = 0.0098) at 24 h and three-fold (42.6 ± 4.39%, *p* < 0.0001) at 48 h, compared to control groups (17.9 ± 0.8%). This increased internalized radioactive fractions correlated positively with the displayed increase in surface-bound radiotracer in treated cells incubated at 4 °C. Overall, this in vitro study demonstrate that [^89^Zr]Zr-cetuximab measured an increase in membranous EGFR after blockade of Src activity in good agreement with results from the western blots.

### [^89^Zr]Zr-cetuximab is specific for tumors expressing EGFR in vivo

**Fig. 2:**
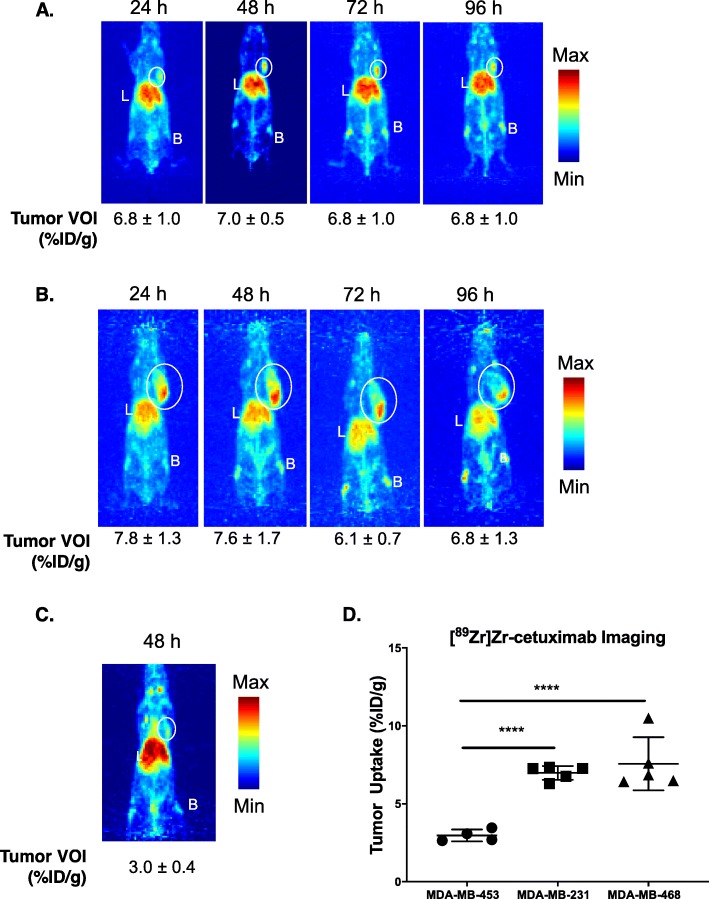
[^89^Zr]Zr-cetuximab PET in EGFR differentially expressing TNBC xenografts. Mice bearing EGFR-high MDA-MB-231 (**a**) or MDA-MB-468 (**b**) and EGFR-low MDA-MB-453 (**c**) tumors imaged with [^89^Zr]Zr-cetuximab over time consistently displayed at least two-fold higher tumor uptake of the radiotracer in the high-expressing xenografts compared to the low EGFR-expressing tumor (**d**). Images are shown as maximum intensity projections. L = liver, B = bone. Tumors are identified by a white circle

The specificity of [^89^Zr]Zr-cetuximab for EGFR was investigated through in vivo imaging using mice bearing different EGFR-expressing TNBC tumors (MDA-MB-468 = MDA-MB-231 > MDA-MB-453). In MDA-MB-231, tumor uptake was 6.8 ± 1.0%ID/g at 24 h p.i. and 7.0 ± 0.4%ID/g at 48 h p.i. Tumor accumulation plateaued at 96 h with 8.7 ± 2.9%ID/g (Fig. 2a). In MDA-MB-468 xenografts, tumor uptake was 7.8 ± 1.3%ID/g at 24 h p.i., 7.6 ± 1.7%ID/g at 48 h p.i. and 6.8 ± 1.2%ID/g at 96 h p.i. (Fig. 2b). Tumor-to-normal tissue ratios for both xenografts showed high contrast at 48 h p.i. (Fig. S2A-B), establishing this time point as the optimal imaging time. Nominal non-specific binding was observed in the low EGFR-expressing MDA-MB-453 tumors (Fig. 2c) compared to MDA-MB-231 (*p* < 0.001) and MDA-MB-468 (*p* < 0.005) (Fig. 2d).

### In vivo monitoring of EGFR with [^89^Zr]Zr-cetuximab post-treatment with dasatinib

**Fig. 3:**
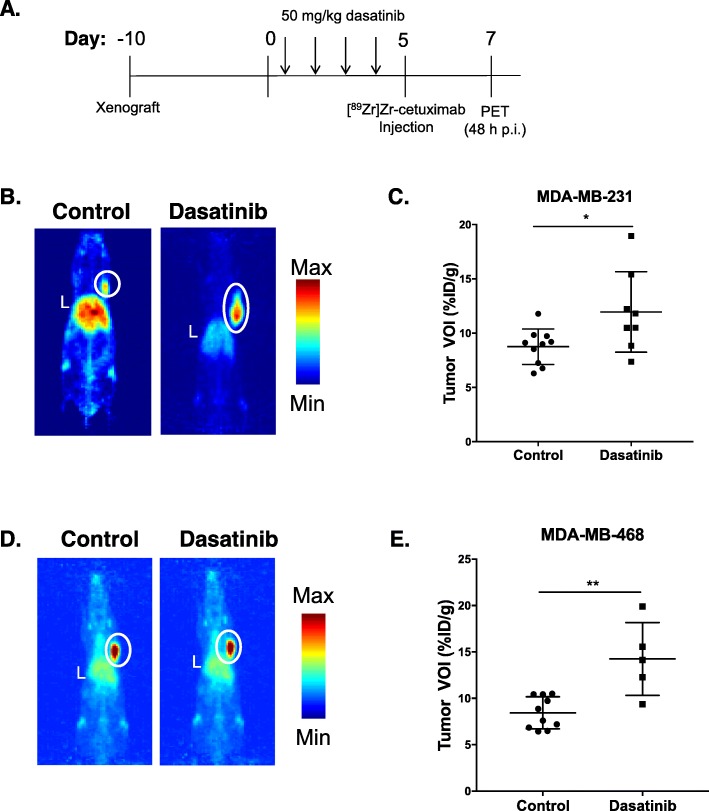
In vivo [^89^Zr]Zr-cetuximab PET imaging tumor uptake after Src inhibition. Mice bearing tumors were treated with dasatinib or vehicle for 5 days before undergoing [^89^Zr]Zr-cetuximab PET imaging on day 7 (**a**). Representative maximum intensity projections (MIPs) of control (left) and dasatinib-treated (right) mice demonstrate higher uptake of [^89^Zr]Zr-cetuximab in treated MDA-MB-231 tumor cohorts (**b**, **c**). MIPs demonstrate higher uptake of [^89^Zr]Zr-cetuximab in treated MDA-MB-468 (right) vs. control (left) tumors at 48 h p.i. **d** and **e** demonstrate higher uptake of the tracer in treated groups compared to control. L = liver. Tumors are identified by a white circle

Tumor-bearing mice treated for  five days with either dasatinib or vehicle (Fig. 3a) were imaged with [^89^Zr]Zr-cetuximab at 48 h p.i. In MDA-MB-231 xenografts (Fig. 3b), [^89^Zr]Zr-cetuximab had higher tumor accumulation in treated vs. control groups (11.9 ± 3.7%ID/g vs. 8.7 ± 1.6%ID/g, *p* = 0.025) (Fig. 3c). Autoradiography of excised tumors demonstrated spatial distribution of the tracer with higher focal uptake observed in treated vs. control tumor sections (Fig. S2C).

Treated MDA-MB-468 tumors (Fig. 3d) exhibited an almost two-fold increase in [^89^Zr]Zr-cetuximab uptake compared to control, untreated tumors (14.2 ± 3.9%ID/g vs. 8.4 ± 1.7%ID/g, *p* = 0.001) (Fig. 3e). Autoradiographic images of excised tumors post-imaging displayed an increase in tracer uptake in dasatinib-treated tumors compared to control untreated tumors (Fig. S2D).

### Ex vivo validation of [^89^Zr]Zr-cetuximab imaging

**Fig. 4:**
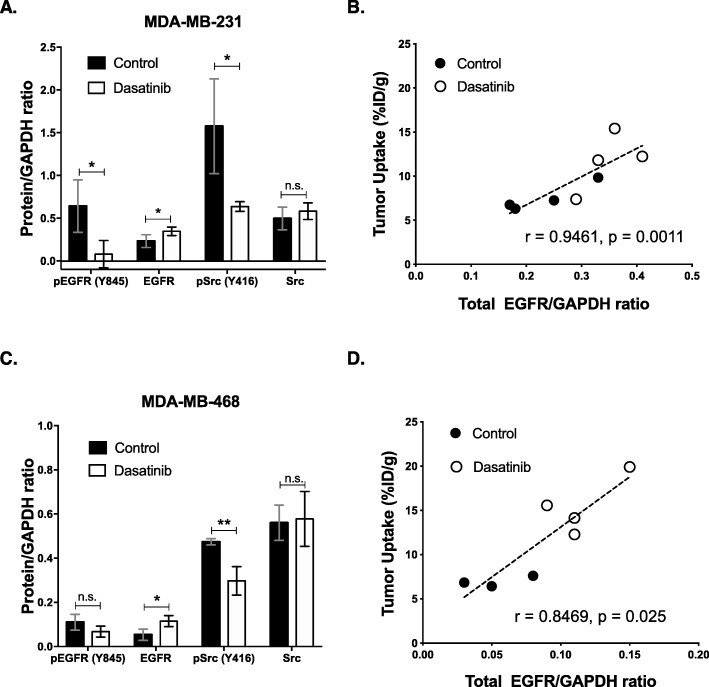
Ex vivo validation of [^89^Zr]Zr-cetuximab PET imaging. Protein/GAPDH ratios obtained from densitometric analysis of western blots of MDA-MB-231 tumors (**a**). Analysis of [^89^Zr]Zr-cetuximab uptake in tumors (%ID/g) plotted against their corresponding total EGFR/GAPDH ratio displayed a direct association of the tumor uptake of the tracer with EGFR expression (**b**). Densitometry analysis of MDA-MB-468 lysates (**c**). A positive correlation was achieved between total EGFR/GAPDH and tumor uptake of the tracer (%ID/g) (**d**)

Western blot densitometry analysis (protein/GAPDH ratio, Fig. 4a, Fig. S3A) of MDA-MB-231 tumors demonstrated higher EGFR levels in treated mice versus untreated cohorts (0.35 ± 0.05 vs. 0.23 ± 0.07, *p* = 0.043). Functional EGFR (pEGFR (Y845)) was mitigated after dasatinib treatment (0.64 ± 0.31 vs. 0.08 ± 0.16, *p* = 0.02). An almost three-fold decrease in pSrc (Y416) expression was displayed in tumors given dasatinib versus vehicle-treated mice (0.64 ± 0.06 vs. 1.6 ± 0.55, *p* = 0.02). Total Src expression was not significantly different (0.50 ± 0.13 vs. 0.58 ± 0.10, *p* = 0.341). A closer look between accumulation of [^89^Zr]Zr-cetuximab within the tumor and total EGFR/GAPDH ratios revealed a strong positive correlation (*r* = 0.9461, *p* = 0.0011) (Fig. 4b).

Immunoblots from the excised MDA-MB-468 tumors further reinforced the tracer readout. A significant increase in total EGFR (0.12 ± 0.03) compared to control tumors (0.05 ± 0.03, *p* = 0.024) was displayed (Fig. 4c, Fig. S3B). pEGFR (Y845) was lower with dasatinib treatment compared to control (0.07 ± 0.03 vs. 0.11 ± 0.4) but was not statistically significant. pSrc (Y416) decreased by two-fold post-treatment (0.30 ± 0.6 vs. 0.49 ± 0.02, *p* = 0.006) while no significant change in total Src protein level after dasatinib treatment (0.56 ± 0.08 vs. 0.58 ± 0.12, *p* = 0.842) was observed. In addition, a strong positive correlation between normalized total EGFR and tumor uptake of the radiotracers was achieved (*r* = 0.8469, *p* = 0.025) (Fig. 4d).

### Effects of combinatorial dasatinib and cetuximab therapy

**Fig. 5:**
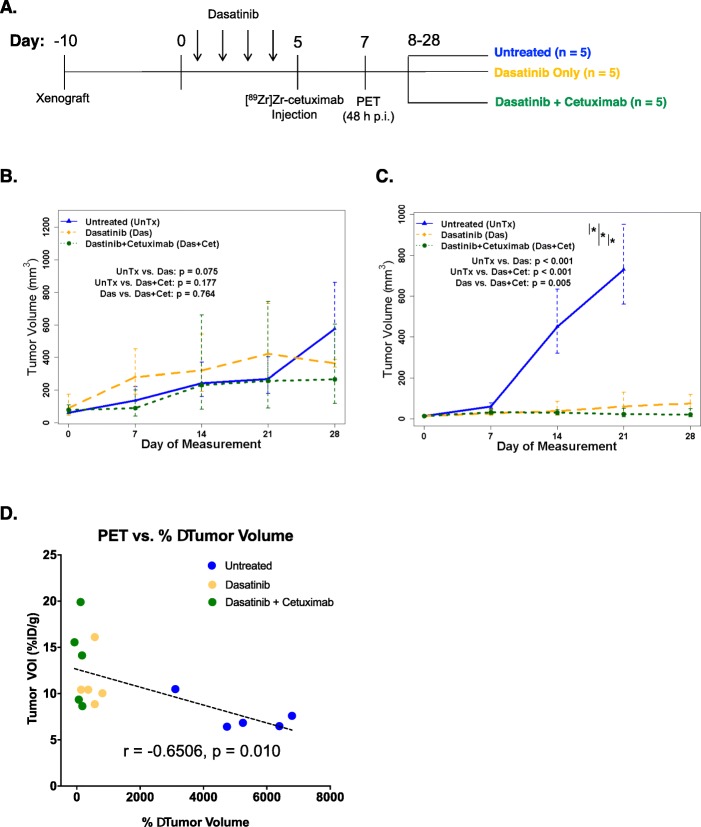
Dasatinib (Das) and cetuximab (Cet) combinatorial treatment after [^89^Zr]Zr-cetuximab PET. The treatment and imaging scheme for tumor-bearing mice employed initial dosing of dasatinib (50 mg/kg, 5×/week, p.o.) or vehicle (UnTx) for 5 days followed by PET imaging on day 7. The mice were then separated into three groups, keeping the vehicle-treated mice till day 28. The other cohorts were continued for treatment with dasatinib alone or in combination with cetuximab (0.2 mg i.p. 2×/week) (**a**). The tumor growth rates were compared using linear mixed-effects models on log-transformed data, and the *p* values were adjusted for multiplicity using Holm’s procedure in MDA-MB-231 (**b**) and MDA-MB-468 (**c**) xenografts. Asterisks indicate significant differences, and the error bars represent 95% confidence intervals. A plot of the tracer uptake vs. %Δ tumor volumes of MDA-MB-468 tumors exhibited a direct correlation with higher PET tracer uptake corresponding to higher tumor regression (**d**)

We then examined whether addition of cetuximab in combination with dasatinib after Src inhibition produces a combinatorial effect. In this longitudinal study, mice treated with dasatinib were stratified into two arms after PET imaging. One group received continuous dasatinib treatment while a second group received dasatinib plus cetuximab (Fig. 5a). The same control group of mice used in the imaging scan was further monitored for tumor progression throughout the study.

In MDA-MB-231 tumor-bearing mice, no tumor response benefit was achieved in both treatment arms (Fig. 5b, Fig. S4A). On the other hand, in MDA-MB-468 tumor-bearing mice, a significant benefit was achieved in mice treated with dasatinib and cetuximab vs. untreated (*p* < 0.001). Moreover, the combinatorial treatment yielded improved treatment outcomes vs. dasatinib (*p* = 0.005) (Fig. 5c, Fig. S4B).

No correlation was derived between [^89^Zr]Zr-cetuximab tumor uptake (VOI) and percentage of change in tumor volume after treatment in MDA-MB-231 (Fig. S4C) (*r* = 0.095, *p* = 0.735). Upon examination of MDA-MB-468 tumors, there was a significant negative correlation between tumor uptake of [^89^Zr]Zr-cetuximab and changes in tumor volume that resulted in slower growth (*r* = − 0.6506, *p* = 0.010) (Fig. 5d).

### Evaluating changes in EGFR localization after dasatinib treatment in TNBC PDX

**Fig. 6:**
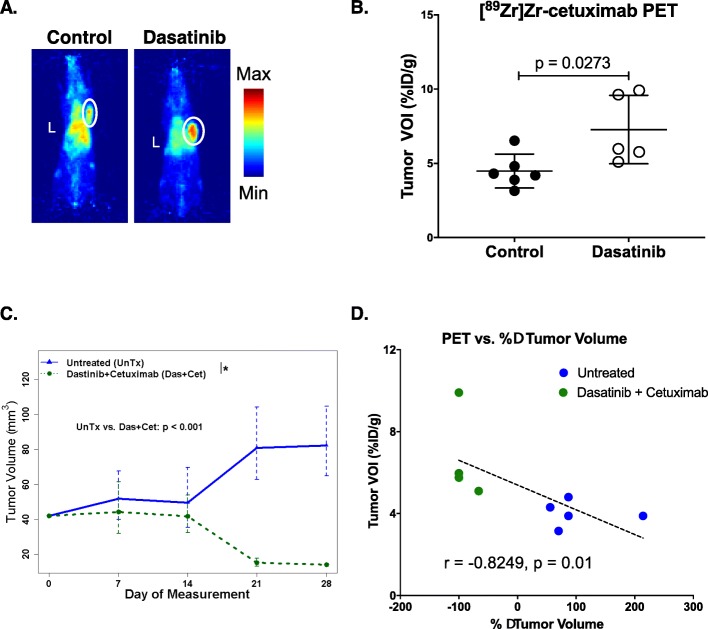
[^89^Zr]Zr-cetuximab imaging monitored TNBC PDX response after dasatinib treatment. PDX-bearing mice treated with dasatinib (right) or vehicle (left) for 5 days were administered [^89^Zr]Zr-cetuximab on the last day of treatment and imaged at 48 h p.i. (**a**). [^89^Zr]Zr-cetuximab tumor VOIs demonstrate higher uptake of the tracer in treated mice compared to control (**b**). The tumor growth rate was compared using linear mixed-effects models on log-transformed data and the *p* values were adjusted for multiplicity using the Holm procedure (**c**). The asterisk indicates a significant difference and the error bars represent 95% confidence intervals. A plot of the tumor uptake of the tracer vs. %Δ tumor volume exhibited a strong, direct association wherein higher the tracer uptake correlated with higher tumor regression (**d**). L = liver. Tumors were identified by a white circle

To assess the clinical significance of our studies, we investigated the effects of dasatinib treatment and the potential of [^89^Zr]Zr-cetuximab to monitor changes in EGFR membrane abundance in an EGFR-expressing TNBC PDX tumor model. Palpable tumors dosed with dasatinib for 5 days had a significantly higher tracer uptake compared to the control untreated arm (7.3 ± 2.3%ID/g vs. 4.5 ± 1.1%ID/g, *p* = 0.027) (Fig. 6a, b). Similar to the established tumor models, the PDX groups were treated with the combinatorial therapy for three additional weeks (Fig. 6c, Fig. S4D). These tumors showed marked volume regression compared to control with three out of four tumors completely disappearing after treatment (*p* < 0.001). A significant, negative correlation between tumor volume changes and tumor uptake of [^89^Zr]Zr-cetuximab was achieved (*r* = − 0.8249, *p* = 0.01) (Fig. 6e).

## Discussion

Recently, zirconium-89 labeled antibodies nimotuzumab [25], imgatuzumab [26], and panitumumab [27, 28], and affibody ZEGFR:2377 [29] were investigated for use in imaging EGFR expression in vivo in addition to [^89^Zr]Zr-cetuximab [30]. All these studies established EGFR as a promising and robust target for immunoPET imaging and targeted radiotherapeutics [31]. Unfortunately, disparities between in vivo EGFR expression and [^89^Zr]Zr-cetuximab PET uptake have been observed [30]. This may be in part due to the compartmentalization of EGFR between the nucleus and non-nuclear compartments [11]. In fact, patients with high nEGFR expression have poor survival and prognosis, particularly in non-small cell lung cancer [8]. The function of Src as a key modulator of EGFR transport to the nucleus is widely accepted [12]. This non-receptor tyrosine kinase was implicated as an important downstream node of cetuximab response pathways [11, 12, 15, 16]. Previous studies tested the causal effects of mitigating Src kinase activity using dasatinib in TNBC with high nEGFR levels. Li et al. demonstrated that treatment of cetuximab-resistant cells with dasatinib resulted in nEGFR loss and increased membrane EGFR expression, which correlated with a re-sensitization to cetuximab treatment [11]. Through in vitro studies, Brand et al. demonstrated concomitant translocation of EGFR to the plasma membrane upon dasatinib treatment, underscoring this pathway as a key strategy to enhance EGFR cell-surface availability for targeted treatments [12]. Taken together, these seminal findings correlate well with our results.

Having proven the specificity of [^89^Zr]Zr-cetuximab for EGFR through in vivo imaging between high and low expressing EGFR TNBC xenografts, we set out to demonstrate that this imaging tracer can guide treatment decisions by assessing re-sensitization of tumors to cetuximab post-Src kinase inhibition. In our hands, increased [^89^Zr]Zr-cetuximab tumor uptake was observed post-dasatinib treatment. This result can only be achieved when more EGFR is accessible on the cell surface for the antibody-based radiotracer to bind. EGFR redistribution from the nucleus to the membrane was evidenced by the enhanced accumulation of the radiotracer on the cell surface upon stopping temperature-mediated endocytosis (Fig. 1e). The concomitant increase in internalization of [^89^Zr]Zr-cetuximab-bound to EGFR validated this finding as EGFR is well known to internalize via clathrin-mediated endocytosis followed by either degradation or recycling to the membrane [21]. Further proof of reduction of nEGFR upon potent mitigation of Src activity was supported by the results from the immunoblots (Fig. 1c, d).

Of note, in vitro total Src levels were degraded versus findings from the in vivo treatment studies. This discrepancy can be attributed to the high IC_50_ (μM) established by us, compared to previous reports that utilized nanomolar concentrations [12, 32] which did not affect its expression. The high dose likely induced pharmacological effects on Src as well as on EGFR. In contrast, the dose (50 mg/kg) administered in vivo, similar to doses employed by other studies [32], did not potentiate Src degradation possibly due to the presence of multiple non-Src targets of dasatinib. Furthermore, the dose may be not be clinically efficacious with dasatinib exposures limited by its pharmacokinetics. Of critical importance is the fact that the in vivo dose more than adequately inhibited phosphorylation of Src—the primary established action of dasatinib. Moreover, Src is a robust protein with many mechanisms that protect against its degradation, justifying our rationale for using pSrc as a readout of dasatinib inhibition.

Tumor uptake of [^89^Zr]Zr-cetuximab significantly correlated with tumor regressions of MDA-MB-468 and PDX cohorts that were given the combined dasatinib and cetuximab treatment Thus, achieving a significant treatment benefit. No improved outcome was displayed in MDA-MB-231 cells. We believe the non-synergistic effect of the combinatorial treatment can be attributed to the Kras mutation status of MDA-MB-231. This limits the predictive potential of the tracer to tumors with only a KRAS wt profile. It is important to note that KRAS mutations are not commonly observed whereas KRAS wt is predominant in ~ 60% of EGFR-amplified TNBC [33, 34]. Taken together, the findings of our study are meaningful as successful treatment outcomes are often associated with tumor regression, which is often pronounced at later stages of treatment. Early detection of response to dasatinib treatment via molecular imaging of its effect on EGFR becomes of paramount importance in this setting.

The Window of Opportunity Trial utilizing dasatinib in operable triple negative breast cancers with nEGFR (NCT02720185) was conceptualized to determine if Src mitigation can prevent nuclear translocation of EGFR in stage I–III TNBC. Its primary outcome seeks to evaluate an increase of at least ~25% membrane EGFR expression from baseline to post-dasatinib treatment. While expression can be evaluated via pathological approaches (e.g., IHC and in situ hybridization) [33], this requires multiple sequential biopsies to obtain tissue specimens with some lesions rendered inaccessible due to its depth and location. The invasiveness of this procedure limits tissue sampling for pre- and post-therapy assessment. With this perspective, our initiative to validate [^89^Zr]Zr-cetuximab as a tool to non-invasively and quantitatively monitor the modifications in cellular distribution of EGFR is timely and useful. [^89^Zr]Zr-cetuximab can monitor changes in cell-surface EGFR expression and/or abundance on a per-lesion basis, providing spatial and temporal information in real time. From the in vivo studies, we have shown that [^89^Zr]Zr-cetuximab uptake increased by at least 1.4-fold after dasatinib treatment. Changes measured by the radiotracer can be utilized to determine outcomes of this patient trial, potentially supporting its use as a tool for monitoring effects of dasatinib treatment on EGFR localization in TNBC.

## Conclusion

 [^89^Zr]Zr-cetuximab PET imaging can be utilized to measure effects of inhibition of Src kinase on EGFR to inform its re-sensitization to cetuximab and guide treatment decisions in KRAS wt TNBC.

## Supplementary information

**Additional file 1: Fig. S1.** [^89^Zr]Zr-cetuximab tracer immunoreactivity in MDA-MB-231 cells (A). Dasatinib IC_50_ values in MDA-MB-231 (B) and MDA-MB-468 (C) cells. Surface-bound radioactivity collected from dasatinib-treated and control untreated cells after incubation at 37 °C.

**Additional file 2: Fig. S2.** Binding of [^89^Zr]Zr-cetuximab displayed as tumor-to-tissue ratios (obtained from image analysis) over time in MDA-MB-231 (A) and MDA-MB-468 (B). Autoradiographs obtained from excised MDA-MB-231(C) and MDA-MB-468 (D).

**Additional file 3: Fig. S3.** Western blots of MDA-MB-231 (A) and MDA-MB-468 (B).

**Additional file 4: Fig. S4.** Overall tumor volumes in MDA-MB-231 (**A**) and MDA-MB-468 (**B**) as represented by the area under the tumor growth curve (AUC), normalized by day. Correlation between [^89^Zr]Zr-cetuximab tumor VOI (%ID/g) and percent change in tumor volume after treatment regimen in MDA-MB-231 (**C**). Overall tumor volumes in PDX measured as AUC. **(D)**. The overall tumor volumes were compared using unpaired t-tests on log-transformed normalized AUCs.

**Additional file 5: Table S1.** Western blot densitometry of pEGFR (Y845), EGFR, pSrc (Y416), and Src proteins in MDA-MB-231 and MDA-MB-468 cell lysates (*n* = 3).

**Additional file 6.** Supplemental Information.

## Data Availability

All data generated or analyzed during this study are included in this published article and its supplementary information files.

## Abbreviations

%ID/g: Percentage of injected dose per gram
^89^Zr: ^89^Zirconium
AUC: Area under the curve
EGFR: Epidermal growth factor receptor
i.p.: Intraperitoneal
IC50: Half maximal inhibitory concentration
immunoPET: Immunopositron emission tomography
nEGFR: Nuclear EGFR
p.i.: Post-injection
PBS: Phosphate-buffered saline
PDX: Patient-derived xenograft
radio-iTLC: Radio-instant thin layer chromatography
s.c.: Subcutaneous
TNBC: Triple negative breast cancer
VOI: Volume of interest

## References

[1] Schneider BP, Winer EP, Foulkes WD, Garber J, Perou CM, Richardson A (2008). Triple-negative breast cancer: risk factors to potential targets. Clin Cancer Res.

[2] Corkery B, Crown J, Clynes M, O’Donovan N (2009). Epidermal growth factor receptor as a potential therapeutic target in triple-negative breast cancer. Ann Oncol.

[3] Nakai K, Hung M-C, Yamaguchi H. A perspective on anti-EGFR therapies targeting triple-negative breast cancer. Am J Cancer Res. 2016;6:1609–23. PMC5004067 27648353

[4] Masuda H, Zhang D, Bartholomeusz C, Doihara H, Hortobagyi GN, Ueno NT (2012). Role of epidermal growth factor receptor in breast cancer. Breast Cancer Res Treat.

[5] Gelmon K, Dent R, Mackey JR, Laing K, McLeod D, Verma S (2012). Targeting triple-negative breast cancer: optimising therapeutic outcomes. Ann Oncol.

[6] Brand TM, Iida M, Li C, Wheeler DL (2011). The nuclear epidermal growth factor receptor signaling network and its role in cancer. Discov Med.

[7] Han W, Lo H-W (2012). Landscape of EGFR signaling network in human cancers: biology and therapeutic response in relation to receptor subcellular locations. Cancer Lett.

[8] Traynor AM, Weigel TL, Oettel KR, Yang DT, Zhang C, Kim K, et al. Nuclear EGFR protein expression predicts poor survival in early stage non-small cell lung cancer. Lung Cancer. 2013;81:138–41. 10.1016/j.lungcan.2013.03.020 PMC3679338 23628526

[9] Lo H-W, Xia W, Wei Y, Ali-Seyed M, Huang S-F, Hung M-C (2005). Novel prognostic value of nuclear epidermal growth factor receptor in breast cancer. Cancer Res.

[10] Lo HW, Hsu SC, Hung MC. EGFR signaling pathway in breast cancers: From traditional signal transduction to direct nuclear translocalization. Breast Cancer Res. Treat. 2006;95:211–8. 10.1007/s10549-005-9011-0 16261406

[11] Li C, Iida M, Dunn EF, Ghia AJ, Wheeler DL. Nuclear EGFR contributes to acquired resistance to cetuximab. Oncogene. 2009;28:3801–13. 10.1038/onc.2009.234 PMC2900381 19684613

[12] Brand TM, Iida M, Dunn EF, Luthar N, Kostopoulos KT, Corrigan KL, et al. Nuclear epidermal growth factor receptor is a functional molecular target in triple-negative breast cancer. Mol Cancer Ther. 2014;13:1356–68. 10.1158/1535-7163.MCT-13-1021 PMC4013210 24634415

[13] Brand TM, Iida M, Wheeler DL. Molecular mechanisms of resistance to the EGFR monoclonal antibody cetuximab. Cancer Biol Ther. 2011;11:777–92. 10.4161/cbt.11.9.15050 PMC3100630 21293176

[14] Brand TM, Iida M, Luthar N, Starr MM, Huppert EJ, Wheeler DL (2013). Nuclear EGFR as a molecular target in cancer. Radiother Oncol.

[15] Wheeler DL, Iida M, Kruser TJ, Nechrebecki MM, Dunn EF, Armstrong EA (2009). Epidermal growth factor receptor cooperates with Src family kinases in acquired resistance to cetuximab. Cancer Biol Ther.

[16] Li C, Iida M, Dunn EF, Wheeler DL. Dasatinib blocks cetuximab- and radiation-induced nuclear translocation of the epidermal growth factor receptor in head and neck squamous cell carcinoma. Radiother Oncol. 2010;97:330–7. 10.1016/j.radonc.2010.06.010 PMC2974772 20667610

[17] van Dijk LK, Boerman OC, Kaanders JHAM, Bussink J (2015). PET Imaging in head and neck cancer patients to monitor treatment response: a future role for EGFR-targeted imaging. Clin Cancer Res.

[18] Viola-Villegas NT, Sevak KK, Carlin SD, Doran MG, Evans HW, Bartlett DW, et al. Noninvasive imaging of PSMA in prostate tumors with ^89^Zr-labeled huJ591 engineered antibody fragments: the faster alternatives. Mol Pharm. 2014;11:3965–73. 10.1021/mp500164r PMC4224519 24779727

[19] Lindmo T, Boven E, Cuttitta F, Fedorko J, Bunn PA (1984). Determination of the immunoreactive function of radiolabeled monoclonal antibodies by linear extrapolation to binding at infinite antigen excess. J Immunol Methods.

[20] McKnight BN, Kuda-Wedagedara ANW, Sevak KK, Abdel-Atti D, Wiesend WN, Ku A, et al. Imaging EGFR and HER3 through ^89^Zr-labeled MEHD7945A (duligotuzumab). Sci Rep Nat Publ Group. 2018;8:9043. 10.1038/s41598-018-27454-6 PMC5998059 29899472

[21] Sigismund S, Argenzio E, Tosoni D, Cavallaro E, Polo S, Di Fiore PP. Clathrin-mediated internalization is essential for sustained EGFR signaling but dispensable for degradation. Dev Cell. 2008;15:209–19. 10.1016/j.devcel.2008.06.012 18694561

[22] Tanaka T, Zhou Y, Ozawa T, Okizono R, Banba A, Yamamura T, et al. Ligand-activated epidermal growth factor receptor (EGFR) signaling governs endocytic trafficking of unliganded receptor monomers by non-canonical phosphorylation. J Biol Chem. 2018;293:2288–2301. 10.1074/jbc.M117.811299 PMC5818182 29255092

[23] Eiblmaier M, Meyer LA, Watson MA, Fracasso PM, Pike LJ, Anderson CJ. Correlating EGFR expression with receptor-binding properties and internalization of ^64^Cu-DOTA-cetuximab in 5 cervical cancer cell lines. J Nucl Med. 2008;49:1472–9. 10.2967/jnumed.108.052316 PMC4277815 18703609

[24] Patel D, Lahiji A, Patel S, Franklin M, Jimenez X, Hicklin DJ, et al. Monoclonal antibody cetuximab binds to and down-regulates constitutively activated epidermal growth factor receptor vIII on the cell surface. Anticancer Res. 2007;27:3355–66. 17970081

[25] Chekol R, Solomon VR, Alizadeh E, Bernhard W, Fisher D, Hill W, et al. ^89^Zr-nimotuzumab for immunoPET imaging of epidermal growth factor receptor I. Oncotarget Impact J. 2018;9:17117–32. 10.18632/oncotarget.24965 PMC5908310 29682209

[26] Pool M, Kol A, Lub-de Hooge MN, Gerdes CA, de Jong S, de Vries EGE, et al. Extracellular domain shedding influences specific tumor uptake and organ distribution of the EGFR PET tracer ^89^Zr-imgatuzumab. Oncotarget Impact J. 2016;7:68111–21. 10.18632/oncotarget.11827 PMC5356542 27602494

[27] Wei L, Shi J, Afari G, Bhattacharyya S. Preparation of clinical-grade ^89^Zr-panitumumab as a positron emission tomography biomarker for evaluating epidermal growth factor receptor-targeted therapy. J Label Compd Radiopharm. 2014;57:25–35. 10.1002/jlcr.3134 PMC3982615 24448743

[28] Bhattacharyya S, Kurdziel K, Wei L, Riffle L, Kaur G, Hill GC (2013). Zirconium-89 labeled panitumumab: a potential immuno-PET probe for HER1-expressing carcinomas. Nucl Med Biol.

[29] Garousi J, Andersson KG, Mitran B, Pichl ML, Stahl S, Orlova A, et al. PET imaging of epidermal growth factor receptor expression in tumours using ^89^Zr-labelled ZEGFR:2377 affibody molecules. Int J Oncol. 2016;48:1325–32. 10.3892/ijo.2016.3369 PMC4777594 26847636

[30] Aerts HJWL, Dubois L, Perk L, Vermaelen P, van Dongen GAMS, Wouters BG, et al. Disparity between in vivo EGFR expression and ^89^Zr-labeled cetuximab uptake assessed with PET. J Nucl Med. 2008;50:123–31. 10.2967/jnumed.108.054312 19091906

[31] Sihver W, Pietzsch J, Krause M, Baumann M, Steinbach J, Pietzsch H-J. Radiolabeled cetuximab conjugates for EGFR targeted cancer diagnostics and therapy. Pharmaceuticals (Basel). 2014;7:311–38. 10.3390/ph7030311 PMC3978494 24603603

[32] Luo F, Barrett YC, Yang Z, Camuso A, McGlinchey K, Wen ML, et al. Identification and validation of phospho-SRC, a novel and potential pharmacodynamic biomarker for dasatinib (SPRYCEL™), a multi-targeted kinase inhibitor. Cancer Chemother Pharmacol. 2008;62:1065–74. 10.1007/s00280-008-0699-5 18301894

[33] Secq V, Villeret J, Fina F, Carmassi M, Carcopino X, Garcia S, et al. Triple negative breast carcinoma EGFR amplification is not associated with EGFR, Kras or ALK mutations. Br J Cancer. 2014;110:1045–52. 10.1038/bjc.2013.794 PMC3929875 24423920

[34] Sánchez-Muñoz A, Gallego E, de Luque V, Pérez-Rivas LG, Vicioso L, Ribelles N, et al. Lack of evidence for KRAS oncogenic mutations in triple-negative breast cancer. BMC Cancer. 2010;10:136. 10.1186/1471-2407-10-136 PMC2868051 20385028

