# Supplementary material for: Cetuximab PET delineated changes in cellular distribution of EGFR upon dasatinib treatment in triple negative breast cancer
Source: Breast Cancer Res. 2020 Apr 15;22:37. doi: 10.1186/s13058-020-01270-1 (PMC7160960; doi:10.1186/s13058-020-01270-1)
Supplement: Supplementary file 1 — Additional file 1: Fig. S1. [89Zr]Zr-cetuximab tracer immunoreactivity in MDA-MB-231 cells (A). Dasatinib IC50 values in MDA-MB-231 (B) and MDA-MB-468 (C) cells. Surface-bound radioactivity collected from dasatinib-treated and control untreated cells after incubation at 37 °C. [file 13058_2020_1270_MOESM1_ESM.pdf]

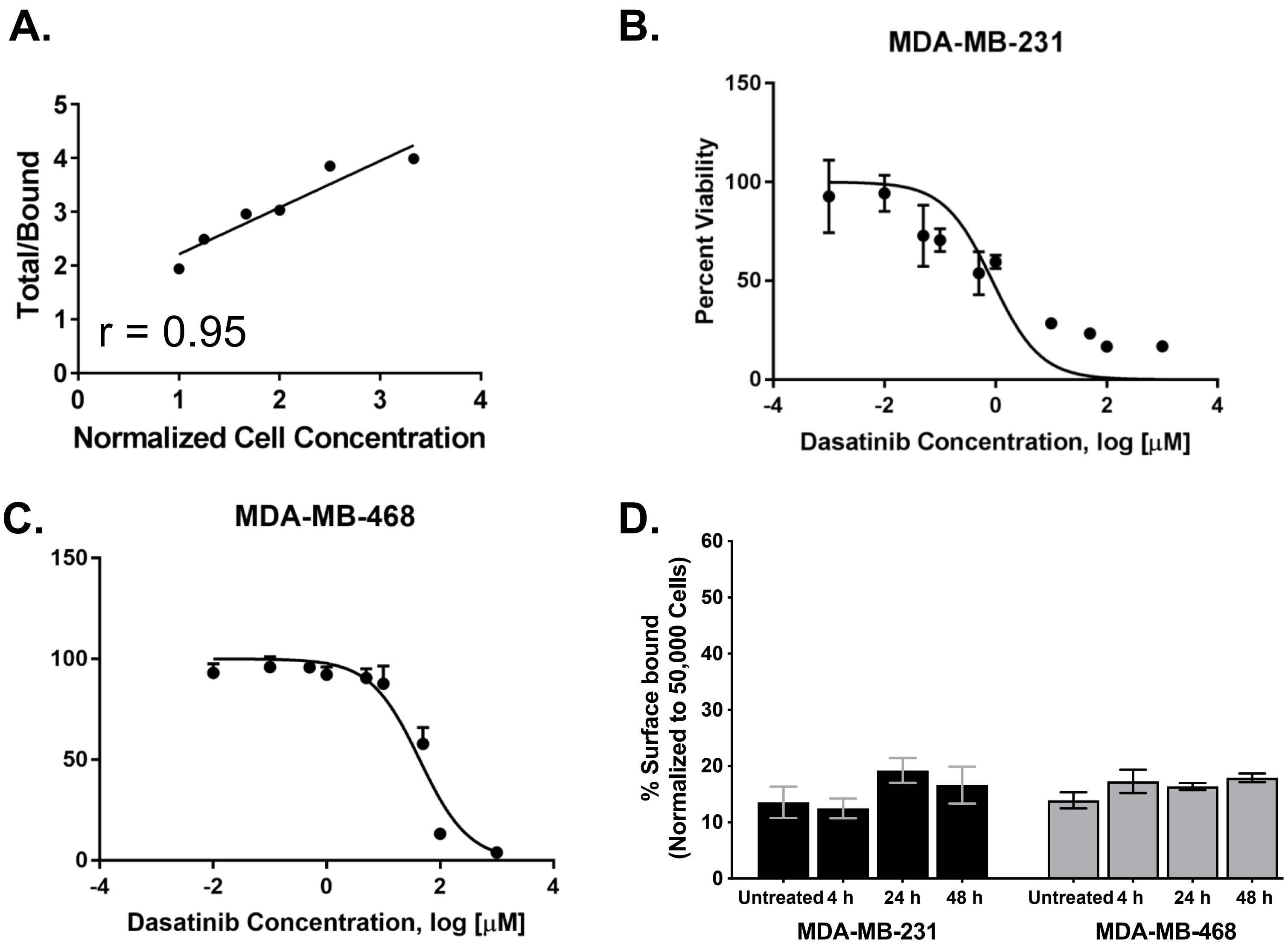

Fig. S1. **[**^{89}Zr]Zr-cetuximab tracer immunoreactivity in MDA-MB-231 cells (**A**). Dasatinib IC_{50} values in MDA-MB-231 (**B**) and MDA-MB-468 (**C**) cells. Surface-bound radioactivity collected from dasatinib-treated and control untreated cells after incubation at 37 °C (**D**).
