# Supplementary material for: Cetuximab PET delineated changes in cellular distribution of EGFR upon dasatinib treatment in triple negative breast cancer
Source: Breast Cancer Res. 2020 Apr 15;22:37. doi: 10.1186/s13058-020-01270-1 (PMC7160960; doi:10.1186/s13058-020-01270-1)
Supplement: Supplementary file 2 — Additional file 2: Fig. S2. Binding of [89Zr]Zr-cetuximab displayed as tumor-to-tissue ratios (obtained from image analysis) over time in MDA-MB-231 (A) and MDA-MB-468 (B). Autoradiographs obtained from excised MDA-MB-231(C) and MDA-MB-468 (D). [file 13058_2020_1270_MOESM2_ESM.pdf]

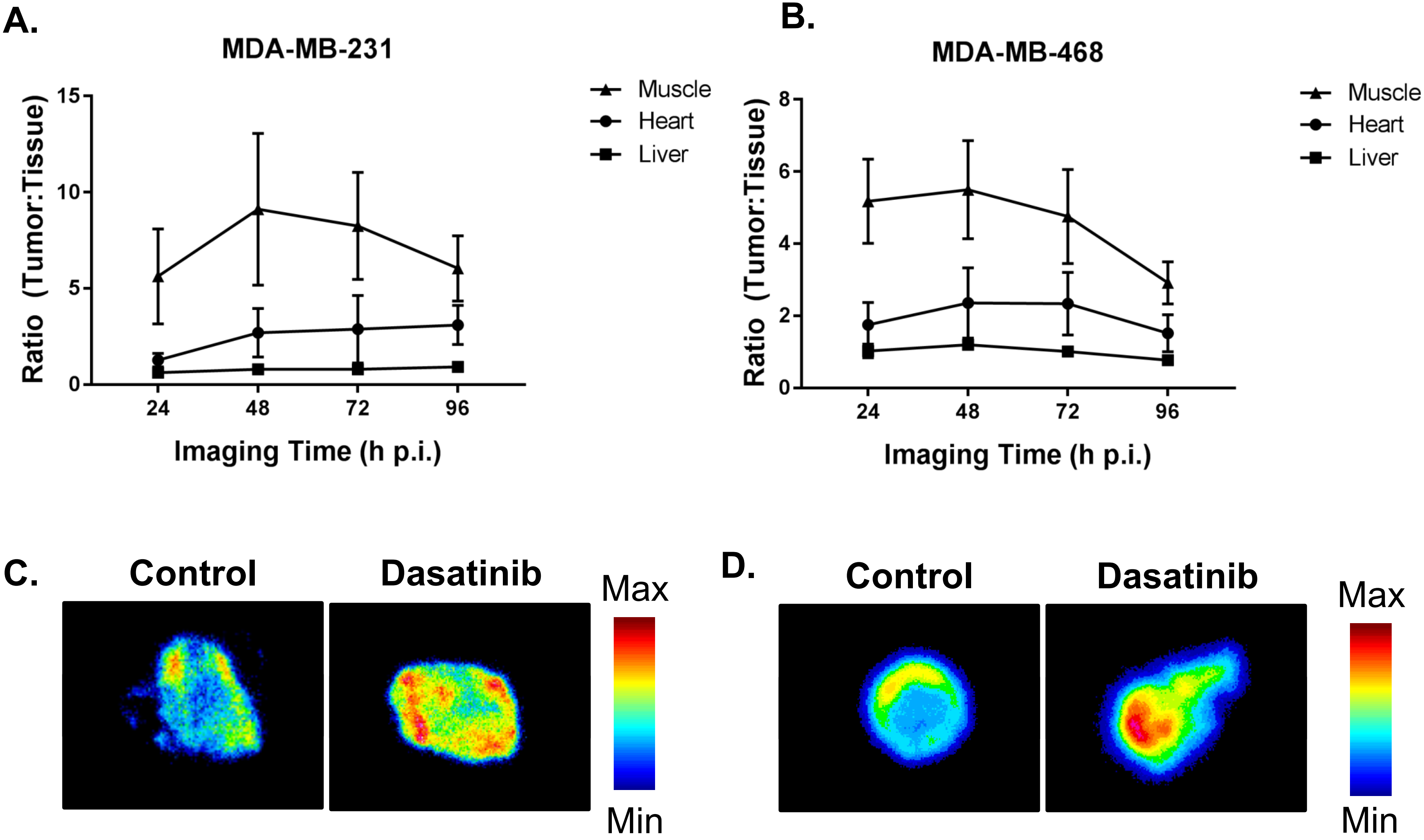

Fig. S2. Binding of [^{89}Zr]Zr-cetuximab displayed as tumor-to-tissue ratios (obtained from image analysis) over time in MDA-MB-231 (**A**) and MDA-MB-468 (**B**). Autoradiographs obtained from excised MDA-MB-231(**C**) and MDA-MB-468 (**D**).
