# Supplementary figures and images for: Cetuximab PET delineated changes in cellular distribution of EGFR upon dasatinib treatment in triple negative breast cancer
Source: Breast Cancer Res. 2020 Apr 15;22:37. doi: 10.1186/s13058-020-01270-1 (PMC7160960; doi:10.1186/s13058-020-01270-1)

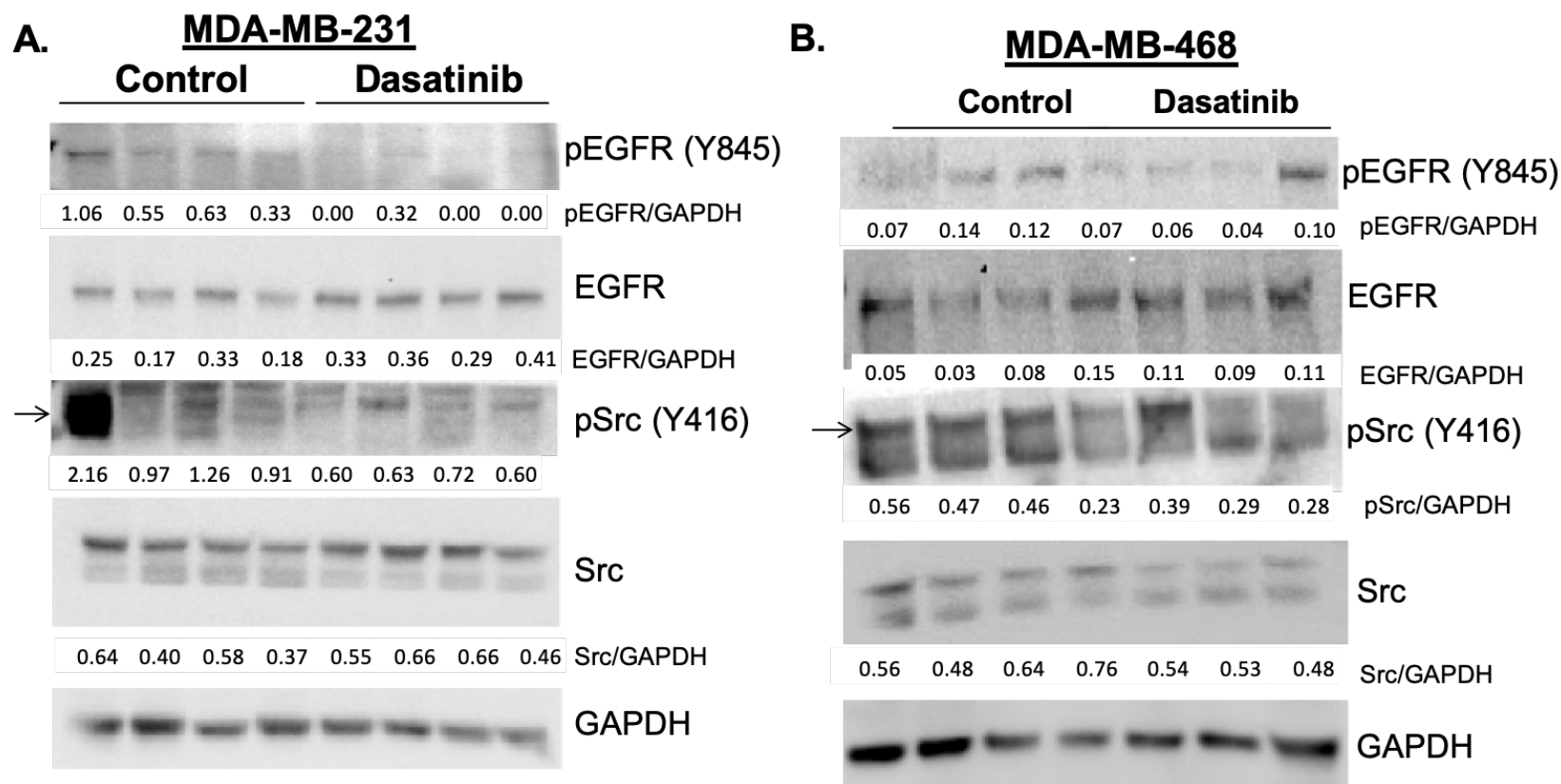

**Fig. S3.** Western blots of MDA-MB-231 (**A**) and MDA-MB-468 (**B**).

Supplement: Supplementary file 3 — Additional file 3: Fig. S3. Western blots of MDA-MB-231 (A) and MDA-MB-468 (B). [file 13058_2020_1270_MOESM3_ESM.pdf]
