# Supplementary material for: Cetuximab PET delineated changes in cellular distribution of EGFR upon dasatinib treatment in triple negative breast cancer
Source: Breast Cancer Res. 2020 Apr 15;22:37. doi: 10.1186/s13058-020-01270-1 (PMC7160960; doi:10.1186/s13058-020-01270-1)
Supplement: Supplementary file 4 — Additional file 4: Fig. S4. Overall tumor volumes in MDA-MB-231 (A) and MDA-MB-468 (B) as represented by the area under the tumor growth curve (AUC), normalized by day. Correlation between [89Zr]Zr-cetuximab tumor VOI (%ID/g) and percent change in tumor volume after treatment regimen in MDA-MB-231 (C). Overall tumor volumes in PDX measured as AUC. (D). The overall tumor volumes were compared using unpaired t-tests on log-transformed normalized AUCs. [file 13058_2020_1270_MOESM4_ESM.pdf]

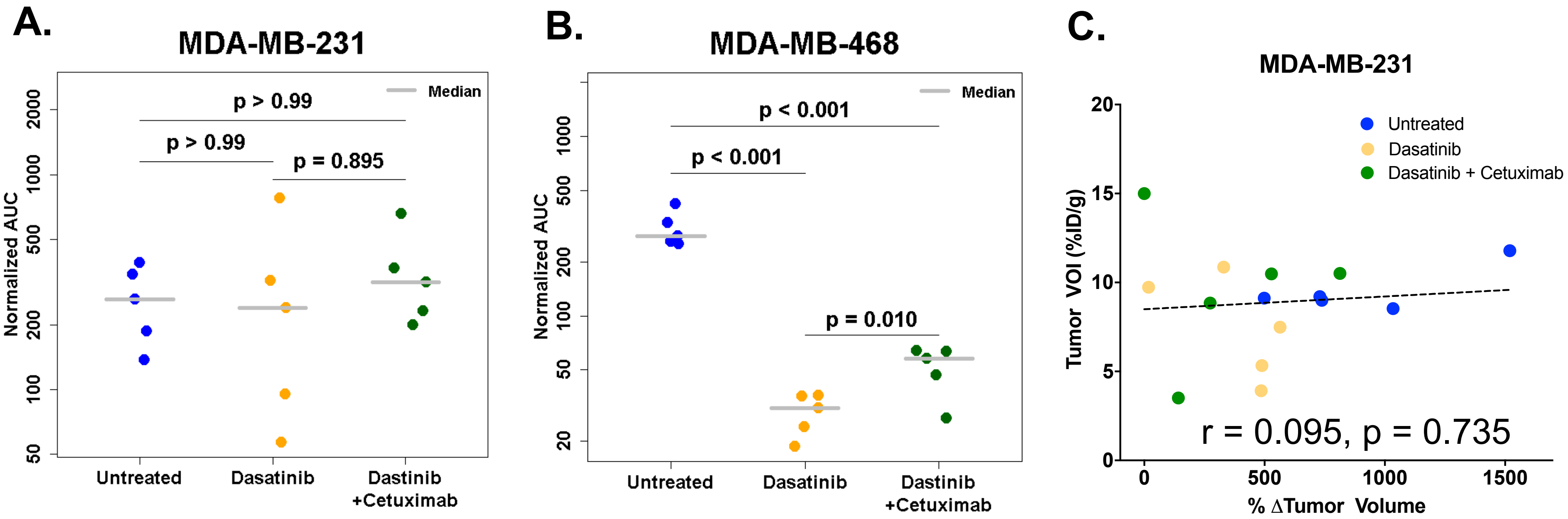

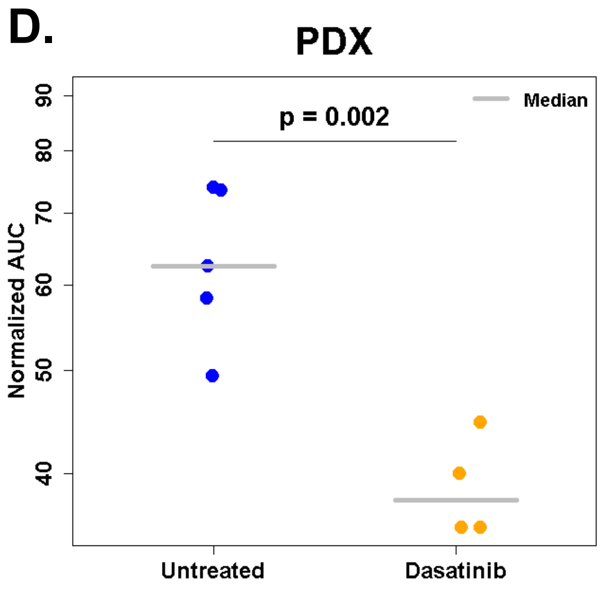

Fig. S4. Overall tumor volumes in MDA-MB-231 (**A**) and MDA-MB-468 (**B**) as represented by the area under the tumor growth curve (AUC), normalized by day. Correlation between [^{89}Zr]Zr-cetuximab tumor VOI (%ID/g) and percent change in tumor volume after treatment regimen in MDA-MB-231 (**C**). Overall tumor volumes in PDX measured as AUC. **(D)**. The overall tumor volumes were compared using unpaired t-tests on log-transformed normalized AUCs.
