# Supplementary material for: Cetuximab PET delineated changes in cellular distribution of EGFR upon dasatinib treatment in triple negative breast cancer
Source: Breast Cancer Res. 2020 Apr 15;22:37. doi: 10.1186/s13058-020-01270-1 (PMC7160960; doi:10.1186/s13058-020-01270-1)
Supplement: Supplementary file 5 — Additional file 5: Table S1. Western blot densitometry of pEGFR (Y845), EGFR, pSrc (Y416), and Src proteins in MDA-MB-231 and MDA-MB-468 cell lysates (n = 3). [file 13058_2020_1270_MOESM5_ESM.pdf]

Protein ratio against GAPDH	MDA-MB-231			MDA-MB-468		
	Control Mean ± S.D.	Dasatinib Mean ± S.D.	P-value	Control Mean ± S.D.	Dasatinib Mean ± S.D.	P-value
**pEGFR (Y845)**	0.83 ± 0.04	0.64 ± 0.02	0.006	1.20 ± 0.04	0.71 ± 0.02	0.004
**EGFR**	0.60 ± 0.11	0.72 ± 0.02	0.403	0.90 ± 0.03	0.98 ± 0.04	0.157
**pSrc (Y416)**	1.6 ± 0.01	0.71 ± 0.04	0.004	1.86 ± 0.01	0.84 ± 0.01	< 0.01
**Src**	0.5 ± 0.03	1.39 ± 0.02	0.007	1.63 ± 0.01	1.33 ± 0.01	0.002

Table S1. Western blot densitometry of pEGFR (Y845), EGFR, pSrc (Y416), and Src proteins in MDA-MB-231 and MDA-MB-468 cell lysates (n=3).
