# Supplementary material for: Cetuximab PET delineated changes in cellular distribution of EGFR upon dasatinib treatment in triple negative breast cancer
Source: Breast Cancer Res. 2020 Apr 15;22:37. doi: 10.1186/s13058-020-01270-1 (PMC7160960; doi:10.1186/s13058-020-01270-1)
Supplement: Supplementary file 6 — Additional file 6. Supplemental Information. [file 13058_2020_1270_MOESM6_ESM.pdf]

Supplemental Information

Cell Lines, Reagents, and Xenografts

Primary [(EGFR-XP, D38B1), (phosphor-Src Tyr416, D49G4), (pEGFR Tyr845, D63B4), (Src, 36D10), (Histone H3, 3H1)] antibodies were purchased from Cell Signaling, GAPDH (365062) was purchased from Santa Cruz, and anti-rabbit and anti-mouse HRP-linked secondary antibodies were purchased from GE (NA934, NA931).

Western Blotting

After dasatinib treatment, cells were lysed on ice using 1× RIPA buffer (Pierce) supplemented with HALT protease and phosphatase inhibitor cocktail (Pierce). Tumors were mechanically lysed using a handheld homogenizer Polytron PE 1200E (VWR) in the same buffer. Total protein was analyzed using Pierce BCA Protein Assay Kit (Thermo Fisher) according to manufacturer's instructions.

Whole cell lysates were prepared in NuPAGE LDS sample buffer (Life Technologies) supplemented with 2-mercaptoethanol (Sigma-Aldrich), and brought up to 15 μL with lysis buffer, and incubated at 95 °C for 5 min. Nuclear extraction was performed using NE-PER Nuclear and Cytoplasmic Extraction Kit (78899, ThermoFisher). Proteins (20 μg) and ladder (Precision Plus, BioRad) were separated on a 4-12% before transfer to Immobilon-P PVDF membrane (Millipore Sigma. Membranes were blocked in 5% non-fat dry milk in TBS (KD Medical)-0.1% Tween20 (Amresco) for 1 h at room temperature. Primary antibodies were diluted 1:1000 in TBST with 0.02% sodium azide and incubated at 4°C over night with gentle rocking before blotting with HRP-linked secondary antibodies (1:1000) in 5% milk-TBST for 2 h at room temperature. Proteins were visualized using

Amersham ECL (GE) and images were collected using a ChemiDoc (BioRad) system. Images were analyzed using Image Lab (BioRad) software and densitometry was calculated using ImageJ software (NIH) following previously described protocol (http://www.yorku.ca/yisheng/Internal/Protocols/ImageJ.pdf).

Briefly, full western blot images were saved as a JPEG and opened with image J and converted to grayscale. Measurements were set to only use mean gray value. Regions of interest were defined with a box manually drawn around the largest band on the western blot, retain a measurement of the band itself, and the blot background either above or below the band. Manually move the frame to the next band and repeat until the entire western blot has measured values. All values were pasted into excel and inverted by subtracting the measured value from 225. The background measurement was then subtracted from the band measurement. This value was then divided by the loading control measurement to retain the ratio.
